# Clinical long-term nocturnal sleeping disturbances and excessive daytime sleepiness in Parkinson’s disease

**DOI:** 10.1371/journal.pone.0259935

**Published:** 2021-12-01

**Authors:** Rocio Del Pino, Ane Murueta-Goyena, Unai Ayala, Marian Acera, Mónica Fernández, Beatriz Tijero, Mar Carmona, Tamara Fernández, Iñigo Gabilondo, Juan Carlos Gómez-Esteban

**Affiliations:** 1 Neurodegenerative Diseases Group, Biocruces Bizkaia Health Research Institute, Barakaldo, Bizkaia, Spain; 2 Department of Neurosciences, University of the Basque Country (UPV/EHU), Leioa, Bizkaia, Spain; 3 Biomedical Engineering Department, Faculty of Engineering, Mondragon Unibertsitatea, Mondragon, Gipuzkoa, Spain; 4 Faculty of Medicine Neurology, University of the Basque Country (UPV/EHU), Leioa, Spain; 5 Cruces University Hospital, Barakaldo, Bizkaia, Spain; 6 Ikerbasque: The Basque Foundation for Science, Bilbao, Spain; Oslo Universitetssykehus, NORWAY

## Abstract

**Objective:**

To prospectively evaluate nocturnal sleep problems and excessive daytime sleepiness (EDS) in Parkinson’s disease (PD) patients, and analyze the influence of motor symptoms, treatment, and sex differences on sleep problems in PD.

**Methods:**

Sleep disturbances of 103 PD patients were assessed with Parkinson’s Disease Sleep Scale (PDSS) and the Epworth Sleepiness Scale (ESS). Student’s t-test for related samples, one-way ANOVA with Tukey’s HSD post hoc test were used to assess group differences. Bivariate correlations and mixed-effects linear regression models were used to analyze the association between clinical aspects and sleep disturbances over time.

**Results:**

At baseline, 48.5% of PD patients presented nocturnal problems and 40% of patients presented EDS. The PDSS and ESS total score slightly improve over time. Nocturnal problems were associated with age and motor impartment, explaining the 51% of the variance of the PDSS model. Males presented less nocturnal disturbances and more EDS than females. Higher motor impairment and combined treatment (L-dopa and agonist) were related to more EDS, while disease duration and L-dopa in monotherapy were related to lower scores, explaining the 59% of the model.

**Conclusions:**

Sleep disturbances changed over time and age, diseases duration, motor impairment, treatment and sex were associated with nocturnal sleep problems and EDS. Agonist treatment alone or in combination with L-dopa might predict worse daytime sleepiness, while L-dopa in monotherapy is related to lower EDS, which significantly affects the quality of life of PD patients.

## Introduction

Circadian rhythm dysfunction is common in neurodegenerative diseases such as Parkinson’s disease (PD). The disruption is characterized by a reduction in the amplitude of the circadian rhythm and could appear even before the onset of cardinal motor symptoms of PD [1]. Sleep problems are among the most common non-motor manifestations in PD. More than 60% of patients present sleep disturbances such as insomnia, restless leg syndrome, sleep fragmentation, nocturnal incontinence, REM sleep behavior disorder, excessive daytime sleepiness (EDS) and/or vivid dreams/hallucinations [2–4]. Sleep disorders significantly impact health-related quality of life in PD, inducing fatigue, tiredness, and worsening motor manifestations and affective symptoms [5–8]. PD-related sleep disturbances do not seem to respond to L-dopa therapy and increase throughout the course of the disease [9], due to age-associated sleep-wake regulation problems and drug-related side effects [6, 10, 11].

Despite the high prevalence and clinical impact of sleep disorders in PD, these symptoms are commonly under-diagnosed and, consequently, untreated [1]. Their adequate identification and characterization over the natural course of the disease is crucial for proper sleep health management. Sleep disorders can be assessed by neurophysiological evaluation such as polysomnography and by questionaries such as the Unified Parkinson’s Disease Rating Scale (UPDRS) or the Parkinson’s Disease Questionnaire (PDQ39), but both questionaries are limited in terms of sleep-related items [12, 13]. Several scales have been developed for screening, quantifying, and monitoring the therapeutic response of sleep disorders in PD. The Parkinson’s Disease Sleep Scale (PDSS) is the most widespread in the clinical practice, and allows to rate the level of sleep disruption to select appropriately targeted therapies [14, 15]. On the other hand, the Epworth Sleepiness Scale (ESS) assesses EDS, a symptom characterized by the inability to stay alert and awake during the day with persistent sleepiness, after apparently adequate or prolonged night-time sleep. EDS affects from 20 to 60% of PD patients, and the underlying pathophysiology is multifactorial [16–19]. According to Marano et al. [20] EDS is associated with other PD problems such as swallowing impairment even in the early stages of the disease.

For the present clinical and prospective study, we used a clinical registry of PD-related sleep disorders collected over 10 years, to evaluate the progression of sleep disorders in PD and their association with demographic, motor, and treatment-related features of patients.

## Materials and methods

### Participants

We performed a prospective analysis of sleep disorders in 103 PD patients over 10 years. PD patients were recruited at outpatient clinics at the Department of Neurology of Cruces University Hospital and were evaluated twice during their regular medical visits occurring at varying timepoints of follow-up (mean time to follow-up 48.5 ± 26.6 months, range 8–125 months). The recruitment period was from 2004 to 2018. Patients were informed about the prospective study and gave their written consent prior to their participation, in accordance with the tenets of the Declaration of Helsinki. The clinical characteristics of patients ranged from newly diagnosed to advanced stage PD patients. PD patients fulfilled the United Kingdom PD Society Brain Bank criteria [21]. The exclusion criteria were as follow: 1) patients with clinical features of atypical parkinsonian syndromes, including dementia with Lewy bodies, multiple system atrophy, progressive supranuclear palsy, corticobasal degeneration or vascular parkinsonism; 2) patients with cognitive impairment, unable to respond appropriately to clinical examination or to complete the scales. This study was approved by the Cruces Clinical Research Ethics Committee.

### Clinical and sleep disorders assessment

All patients were evaluated during their regular clinical visit by an experienced neurologist in the field of movement disorders. A semi-structured demographic questionnaire, date of diagnosis, UPDRS, PD-related treatments, and Levodopa Equivalent Daily Dose (LEDD) were recorded at each visit [22]. Patients were subdivided according to treatment type (L-dopa in monotherapy, agonists in monotherapy, and combined therapy).

Patients were classified as tremor dominant (TD) or rigid akinetic (RA) using the numerical ratio derived from a patient’s mean tremor score and mean akinetic-rigidity score, both measured using UPDRS items [23]. Tremor was assessed using seven-items (resting tremor of head and each limb and postural tremor of hands). The 14 items to assess akinetic-rigidity were passive range of motion rigidity of neck and extremities, hand movements, finger taps, arising from a chair, posture, gait, postural instability, and bradykinesia. The mean of each scale was calculated, and the ratio (mean TD/mean RA score) was determined. Based on this method, RA subjects had a ratio <0.8, whereas TD subjects had a ratio >1.0.

The PDSS quantifies sleep disturbances in patients [14] through 15 items, scored from 0 (always) to 10 (never). A total PDSS≤82 were considered to have sleep disturbances [24, 25]. The ESS is an 8-item self-administered questionnaire which provides the subject’s general level of daytime sleepiness scored from 0 (never doze) to 3 (high chance of dozing) [26]. The total score ranges from 0 to 24, higher scores reflect greater sleep propensity. A total ESS score >10 represents EDS [24, 25].

### Statistical analysis

Statistical analyses were carried out with IBM SPSS Statistics for Windows, (v.21.0). Group differences for continuous variables were analyzed with two-tailed Student’s t-test for related samples and one-way ANOVA with Tukey’s HSD post-hoc test for multiple group comparisons. Bivariate Pearson correlations were computed to explore at baseline and at follow-up the relationship between the sleep disturbance (PDSS and ESS) with motor symptoms (UPDRS-III), disease duration, age, and drug treatment. Mixed-effects linear regression models were used, fitting separate models for nocturnal problems (PDSS total score and PDSS items) and daytime sleepiness (ESS Total score and ESS items) by maximum likelihood, using MATLAB, to rate the sleep changes over time. We used “time to follow-up (years)”, “age at baseline”, “sex”, “diseases duration at baseline”, “UPDRS-III”, and “treatment” [L-dopa in monotherapy, agonist in monotherapy, combined treatment (yes/no)] into the models as fixed-effects and a random intercept for “subjects” (ID). The baseline and follow-up variables were both included to analyze how the variables changed according to time. For each analysis, different models, including all possible variables combinations, were constructed and the model with the smallest Akaike information criterion (AIC) value was selected as the optimal mixed-effect model. Optimal model´s validity was assessed performing likelihood ratio tests compared to the null models [27].

## Results

### Demographics and clinical features

**Fig 1:**
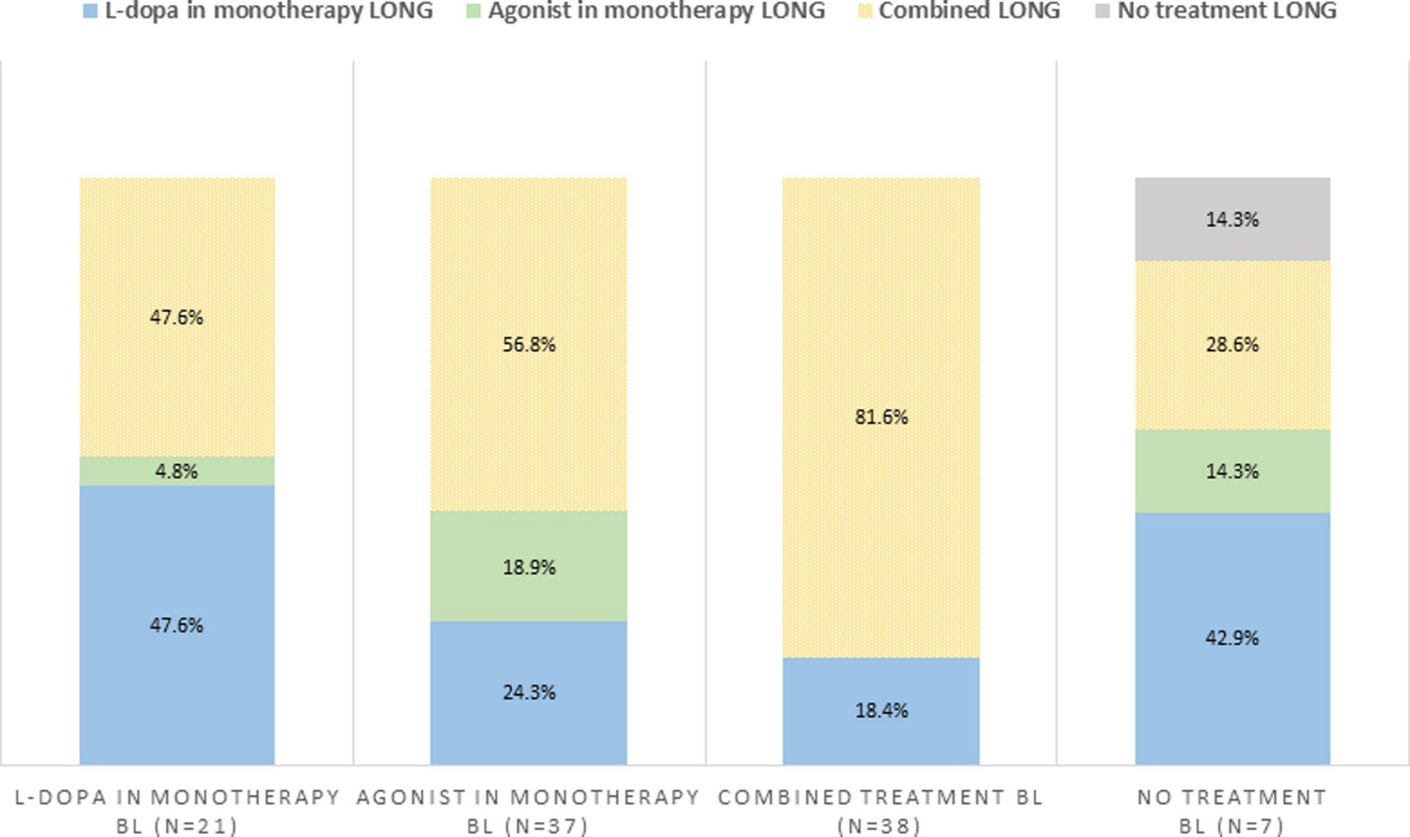
Change of treatment from baseline to follow-up. Abbreviations: BL: Baseline; LONG: Longitudinal, treatment at follow-up.

The general characteristics of study participants at baseline and at follow-up are shown in Table 1. The 52.4% of patients were males, the mean (SD) age of patients was 64.24 (9.16) years, the duration of the disease was 3.94 (4.20) years at baseline. Most patients were RA (92.8%) and were on dopaminergic agonist treatment in monotherapy (35.9%) or in combined therapy (36.9%), while 20.4% of patients were under L-dopa in monotherapy and 6.8% were not taking medication at baseline. At follow-up, most patients were on combined therapy (62.1%), 28.2% were on L-dopa in monotherapy, 8.7% agonist in monotherapy, and just 1% of patients were without treatment. Fig 1 shows the percentage of patients that continued with the same treatment at follow-up and the percentage of patients that changed their treatment.

**Table 1:** Demographical and PD-related clinical features.

	Baseline	Follow-up	Statistics	
	PD patients (n = 103)	PD patients (n = 103)	*t*	r
Age (years)	64.24 (9.16)	68.39 (9.26)	-18.30**	-
Disease duration (years)	3.94 (4.20)	8.32 (4.99)	-17.23**	-
UPDRS I	2.22 (1.91)	2.85 (1.96)	-3.29*	.49**
UPDRS II	11.01 (5.36)	14.08 (6.06)	-4.96**	.40**
UPDRS III	23.98 (9.04)	32.29 (9.89)	-8.63**	.47**
UPDRS IV	2.74 (2.78)	4.01 (3.10)	-3.96**	.39**
Treatment (mg)				
L-dopa in monotherapy	n = 21, 469.54 (323.16)	n = 29, 606.89 (276.08)	-	-
Agonists in monotherapy	n = 37, 150.43 (173.79)	n = 9, 124.22 (50.34)	-	-
Combined treatment	n = 38, 657.00 (256.54)	n = 64, 739.43 (381.62)	-	-
No treatment	n = 7	n = 1	-	-
PDSS total score	112.07 (16.40)	114.80 (14.54)	-1.78	.50**
ESS total score	8.24 (4.45)	8.26 (4.41)	-.04	.50**

*p < .01;

** *p* < .001. The results are displayed as mean (standard deviation), except for number of patients under dopaminergic agonist treatment (n, %). Longitudinal group differences were analyzed with Student’s t test. Pearson correlations analyses were carried out between the variables at baseline and follow-up. Abbreviations: ESS: Epworth Sleepiness Scale; PDSS: Parkinson Disease Sleep Scale; Treatment: Levodopa Equivalent Daily Dose (mg); UPDRS: Unified Parkinson’s Disease Rating Scale.

Regarding motor impairment, there were statistically significant differences between baseline and follow-up assessment, showing deterioration of clinical characteristics of the disease measured with the UPDRS (I-IV).

Focusing on sleep disturbance, 48.5% presented nocturnal problems (score below 5 on at least one of the PDSS items), 3.9% presented moderate to severe sleep problems (PDSS≤ 82), and 40% of patients presented EDS (ESS>10) at baseline. At follow-up, only 45% of patients and 44.4% of patients presented more nocturnal problems (PDSS total score) and EDS (ESS total score), respectively.

At baseline, lower PDSS score correlated with higher motor impairment (r = -.28, p = .004), and more RA symptoms (r = -.26, p = .008), while ESS positively correlated with dopaminergic agonist treatment (r = .25, p = .011).

### Rate of change in sleep disturbance and daytime sleepiness over time

**Fig 2:**
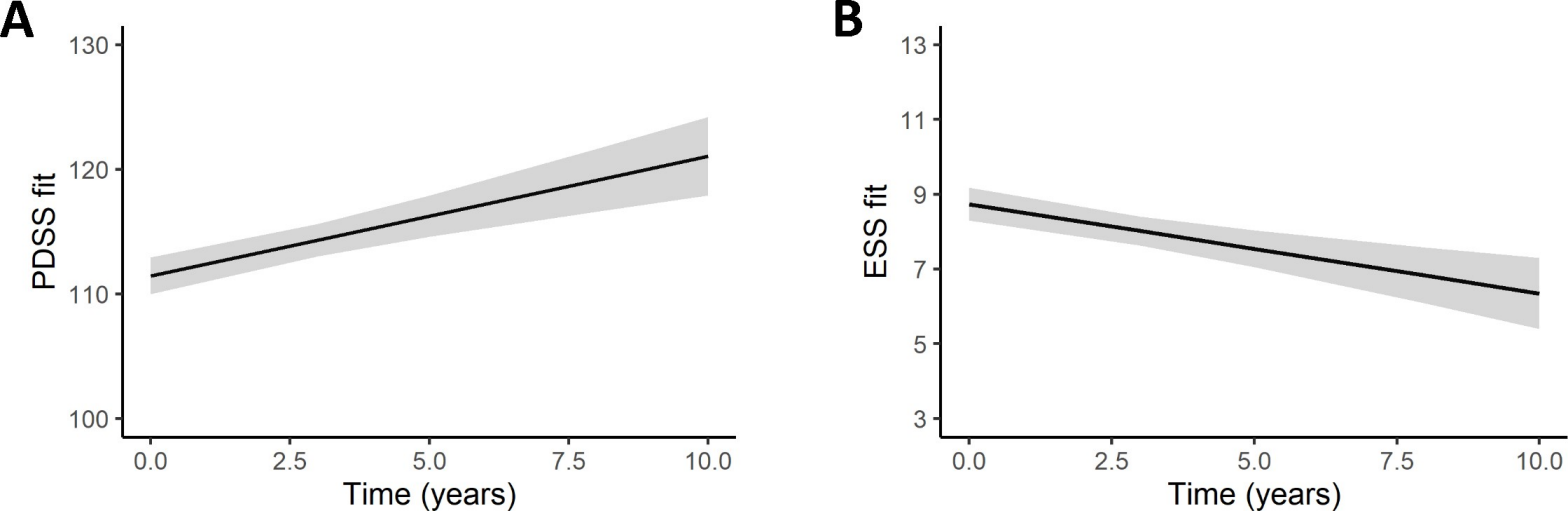
Progression of nocturnal sleeping disturbances and excessive daytime sleepiness in Parkinson’s disease over time. Parameter estimates from linear mixed models are plotted. Lines represent the mean and shadowed grey background the SE of the estimation. Abbreviations: ESS, Epworth Sleepiness Scale total score; PDSS, Parkinson’s disease Sleeping Scale total score.

The optimal regression mixed models for PDSS and ESS are shown in Table 2 and represented in Fig 2. The regression linear mixed analysis was also performed by items of PDSS and ESS, and the models with the lowest AIC value and their corresponding coefficient of determination are represented in Table 3. Additionally, Table 4 summarizes which factors were significantly associated with changes in individual items of PDSS and ESS over time.

**Table 2:** Regression linear mixed model for PDSS and ESS Total score.

Mixed model	β	SE	t	df	Lower	upper
**PDSS Total score model**						
Intercept	134.41	9.05	14.84***	201	116.56	152.26
Time to follow-up (years)	0.95	0.36	2.62**	201	0.23	1.67
Age (years)	-0.27	0.13	-1.98*	201	-0.55	-0.00
UPDRS III (total score)	-0.25	0.11	-2.24*	201	-0.47	0.03
**ESS Total score model**						
Intercept	4.77	1.50	3.22***	195	1.85	7.69
Time to follow-up (years)	-0.22	0.10	-2.10**	195	-0.43	-0.01
UPDRS III (total score)	0.07	0.03	2.30**	195	0.01	0.14
Disease duration (years)	-0.19	0.10	-2.09**	195	-0.37	-0.01
L-dopa monotherapy	-2.50	0.70	-3.57***	195	-3.89	-1.11
Combined therapy	3.77	1.39	2.72**	195	1.03	6.51

Fixed effects coefficient (95% CI).

* < .05;

** < .01;

*** < .001. Abbreviations: ESS: Epworth Sleepiness Scale; PDSS: Parkinson Disease Sleep Scale; UPDRS: Unified Parkinson’s Disease Rating Scale. L-dopa monotherapy and Combined therapy are dichotomic variables (yes/no).

**Table 3:** Linear mixed-effect models selected with the lowest AIC value.

Linear mixed-effect models	AIC	R^2^
**PDSS Total score** ~ 1 + time to follow-up + age at baseline+ UPDRS-III + (1 | id)	1684	.51
**PDSS Item 7** ~ 1 + time to follow-up + UPDRS-III + (1 | id)	756	.45
**PDSS Item 8** ~ 1 + time to follow-up + age at baseline + UPDRS-III + (1 | id)	881	.40
**PDSS Item 9** ~ 1 + time to follow-up + age at baseline + UPDRS-III + (1 | id)	930	.40
**PDSS Item 12** ~ 1 + time to follow-up + sex (males) + treatment (agonists in monotherapy) + (1 | id)	841	.33
**PDSS Item 13** ~ 1 + time to follow-up + (1 | id)	828	.38
**PDSS Item 14** ~ 1 + time to follow-up + age at baseline + sex (males) + (1 | id)	890	.33
**ESS Total score** ~ 1 + time to follow-up + UPDRS-III + disease duration + treatment (L-dopa in monotherapy and combined therapy) + (1 | id)	1146	.59
**ESS Item 1** ~ 1 + time to follow-up + treatment (L-dopa in monotherapy, agonists in monotherapy, and combined therapy) + (1 | id)	588	.45
**ESS Item 2** ~ 1 + time to follow-up + UPDRS III + sex (males) + disease duration + treatment (agonists in monotherapy and combined therapy) + (1 | id)	591	.28
**ESS Item 3** ~ 1 + time to follow-up + UPDRS III + disease duration + treatment (L-dopa in monotherapy) + (1 | id)	567	.12
**ESS Item 4** ~ 1 + time to follow-up + sex (males) + treatment (L-dopa in monotherapy and combined therapy) + (1 | id)	546	.43
**ESS Item 5** ~ 1 + time to follow-up + sex (males) + (1 | id)	598	.31
**ESS Item 7** ~ 1 + time to follow-up + UPDRS III + (1 | id)	597	.26
**ESS Item 8** ~ 1 + time to follow-up + sex (males) + treatment (L-dopa in monotherapy) + (1 | id)	79	46

Abbreviations: AIC: Akaike information criterion; ESS: Epworth Sleepiness Scale; PDSS: Parkinson Disease Sleep Scale; UPDRS: Unified Parkinson’s Disease Rating Scale. Treatment (L-dopa in monotherapy, Agonist in monotherapy, and Combined therapy) is a dichotomic variable (yes/no).

**Table 4:** Rate of changes in sleep disturbance and daytime sleepiness items based on linear mixed models analysis.

	PDSS items	ESS items
**Time to follow-up (points/year)**	↑ Item 13 (β = 0.18, SE = 0.04, t_(201)_ = 3.99)***	
**Age at baseline (points/year)**	↓ Item 8 (β = -0.08, SE = 0.02, t_(201)_ = -4.57)***	-
	↓ Item 9 (β = -0.05, SE = 0.02, t_(200)_ = -2.24)*	
	↓ Item 14 (β = -0.04, SE = 0.02, t_(202)_ = -1.99)*	
**Sex (males)**	↑ Item 12 (β = 1.14, SE = 0.29, t_(202)_ = 3.81)***	↑ Item 2 (β = 0.50, SE = 0.16, t_(194)_ = 3.13)***
	↑ Item 14 (β = 0.74, SE = 0.34, t_(202)_ = 2.20)*	↓ Item 4 (β = -0.36, SE = 0.15, t_(195)_ = -2.40)**
		↑ Item 5 (β = 0.50, SE = 0.17, t_(199)_ = 2.80)**
		↑ Item 8 (β = 0.14, SE = 0.05, t_(198)_ = 2.73)**
**Disease duration (points/year)**	-	↓ Item 2 (β = -0.05, SE = 0.02, t_(194)_ = -2.48)**
		↓ Item 3 (β = -0.04, SE = 0.01, t_(195)_ = -2.26)*
**UPDRS III (points/UPDRS III score)**	↓ Item 7 (β = -0.03, SE = 0.01, t_(202)_ = -3.00)**	↑ Item 2 (β = 0.02, SE = 0.01, t_(194)_ = 2.08)*
	↓ Item 8 (β = -0.03, SE = 0.01, t_(201)_ = -2.3)*	↑ Item 3 (β = 0.02, SE = 0.01, t_(198)_ = 2.67)**
	↓ Item 9 (β = -0.06, SE = 0.02, t_(200)_ = -3.16)***	↑ Item 7 (β = 0.02, SE = 0.01, t_(198)_ = 2.80)**
**Treatment**	-	↓ Item 1 (β = -0.66, SE = 0.18, t_(197)_ = -3.68)***
** L-dopa monotherapy**		
		↓ Item 3 (β = -0.53, SE = 0.16, t_(195)_ = -3.23)**
		↓ Item 4 (β = -0.55, SE = 0.16, t_(195)_ = -3.44)***
		↓ Item 8 (β = -0.09, SE = 0.05, t_(198)_ = -1.98)*
** Agonists monotherapy**	↑ Item 12 (β = 0.99, SE = 0.33, t_(202)_ = 3.00)**.	↑ Item 1 (β = 0.74, SE = 0.37, t_(197)_ = 2.02)*
		↑ Item 2 (β = 0.97, SE = 0.37, t_(194)_ = 2.62)**
** Combined therapy**	-	↑ Item 1 (β = 1.07, SE = 0.35, t_(198)_ = 3.03)**
		↑ Item 2 (β = 1.16, SE = 0.36, t_(194)_ = 3.21)***
		↑ Item 3 (β = 0.70, SE = 0.34, t_(195)_ = 2.05)*
		↑ Item 4 (β = 0.55, SE = 0.17, t_(195)_ = 3.24)***

Fixed effects coefficients (95% CIs).

* < .05;

** < .01;

*** < .001. Note: Lower PDSS scores and higher ESS scores means worse sleep disturbance. Red arrows mean worse disturbances and green arrows means less disturbances. The β-values are the outputs of the linear mixed models regression. They quantify how the variables affect to the PDSS and ESS scores per item. Abbreviations: ESS: Epworth Sleepiness Scale; PDSS: Parkinson Disease Sleep Scale; UPDRS: Unified Parkinson’s Disease Rating Scale. Treatment (L-dopa monotherapy, Agonists monotherapy, and Combined therapy) is a dichotomic variable (yes/no).

Sleep problems assessed by the PDSS total score slightly improved over time. The PDSS total score increased 0.95 points/year from baseline visit to follow-up. While age (-0.27 points/year) and motor impairment (-0.25 points/UPRDS III score) were associated with more sleep disturbances over time, showing more nocturnal disturbance in older people and in patients with greater motor impairment. These variables explained the 51% of the variance of the model.

Regarding the ESS total score model, patients presented less daytime sleepiness (-0.22 points/year) at follow-up. Motor impairment increased 0.07 ESS points/UPDRSIII score while disease duration was associated with a decrease of 0.19 ESS points/disease duration (years). L-dopa in monotherapy decreased ESS total score (-2.50 points), whereas combined therapy increased it (3.77 points). Motor impairment and combined treatment were associated with worse EDS over time, whereas disease duration and L-dopa in monotherapy was related with better ESS total score. The variables time to follow-up, UPDRS III, disease duration and treatment explained 59% of the variance of the daytime sleepiness model.

Analyzing the rate of change by PDSS items over time, we found that the following variables explained between 33% to 59% of the variance of nocturnal problems: time to follow-up (years), age at baseline, sex (males), UPDRS III score, and treatment (agonist in monotherapy). After a deeper analysis of PDSS by items (Table 4), we found that PD patients experienced less tremor on waking (PDSS item 13, 0.18 points) each year from baseline visit. Male sex was associated with less painful postures (PDSS item 12, 1.14 points) and felt less tired and sleepy after waking (PDSS item 14, 0.74 points) over time. Both, older age and higher motor impairment at baseline, were associated with a worsening of nocturnal sleep problems, particularly, with urine incontinence, hallucination, or tiredness. Focusing on treatment, agonist in monotherapy was associated with less painful posturing of extremities at wake up (PDSS item 12, 0.99 points).

Regarding the daytime sleepiness analyzed by ESS items (Table 4), male sex, disease duration, UPDRS III score, and treatment (L-dopa in monotherapy, agonist in monotherapy, and combined therapy) explained between 12% to 46% of the variance. Males sex was associated with worsening of daytime sleepiness watching TV (ESS item 2, 0.50 points), lying down resting (ESS item 5, 0.50 points), and in a car stopped for a few minutes in traffic (ESS item 8, 0.14 points) over time, while female sex was only associated with worsening of doziness in a car over time (ESS item 4, -0.36 points). Higher motor impairment and/or being under agonist in monotherapy or combined therapy were associated with an exacerbation of doziness while sitting and reading, watching TV, sitting inactive in a public, as a passenger in a car, and sitting quietly after lunch. On the other hand, disease duration (years) and/or being under L-dopa treatment in monotherapy was associated with less daytime sleepiness such as less doziness sitting, whether active or inactive.

### Effects of dopaminergic treatment on sleep motor disturbances at follow-up

**Fig 3:**
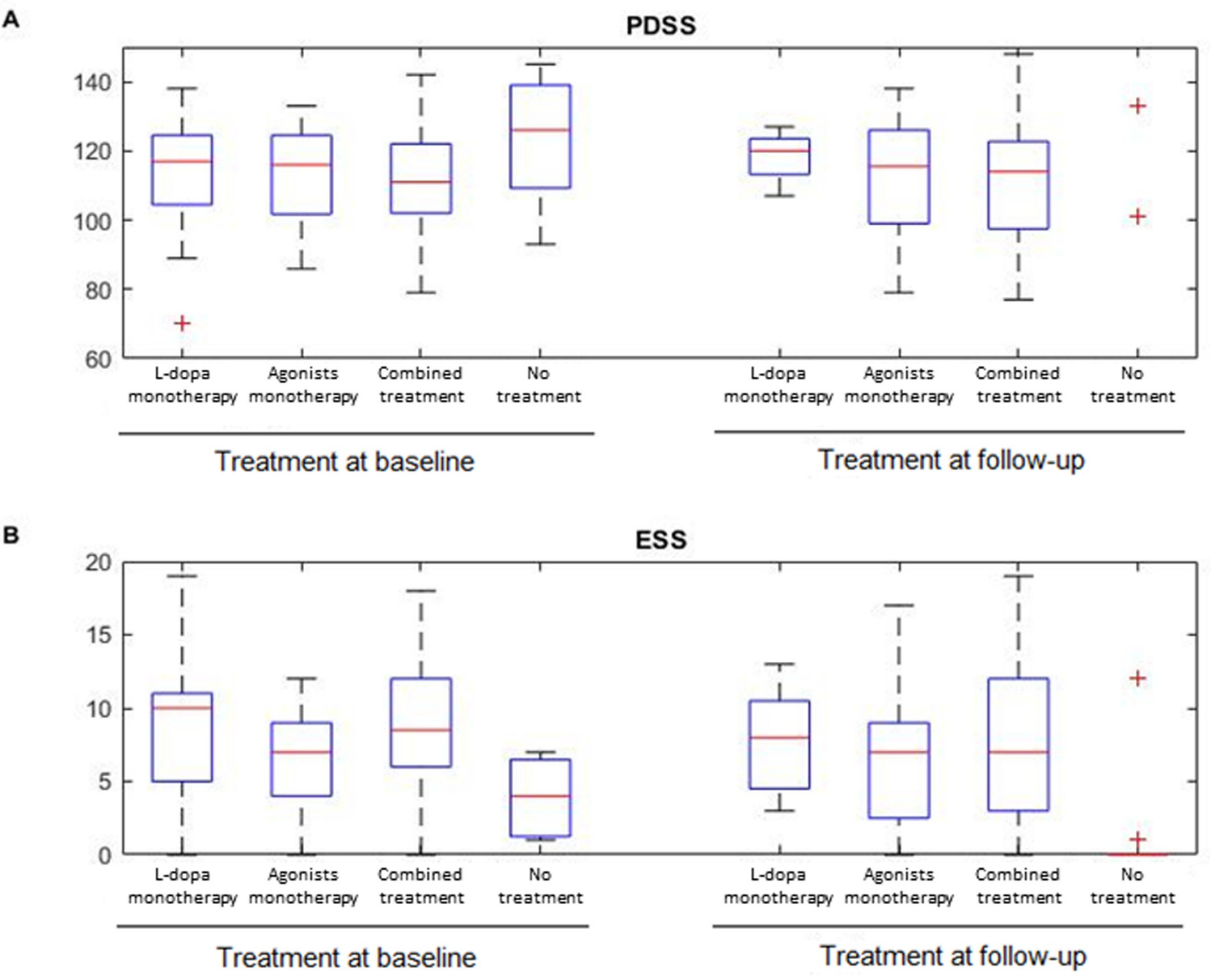
Differences between PDSS and ESS under different treatment. **A.** Scores of the PDSS of patients under dopaminergic treatment at baseline and at follow up. **B.** Scores of the ESS of patients under dopaminergic treatment at baseline and at follow up.

Regarding treatment, we found statistically significant differences in the EDS (baseline: F(3,96) = 4.71, p = .004, η^2^ = .13; follow-up: F(3,98) = 3.25, p = .025, η^2^ = .09), depending on the treatment (Fig 3). Post-hoc analyses revealed that patients with combined therapy presented more EDS than patients under L-dopa in monotherapy at baseline while patients with agonist in monotherapy and combined therapy presented higher scores at baseline and at follow-up compared with patients without treatment. No statistical differences were found in PDSS between treatment.

## Discussion

The present prospective and clinical study investigated nocturnal sleep disturbances and EDS over time in patients with PD, and the potential influence of age, disease duration, motor symptoms, dopaminergic treatment, and sex on sleep problems. Nocturnal sleep problems and EDS slightly improved over time, although older patients with higher motor impairment experienced worsening of sleep problems, and being male, presenting higher motor impairment and being under agonist treatment or combined therapy (agonist and L-dopa) contributed to more EDS at follow-up. Disease duration and L-dopa in monotherapy were associated with decreased hypersomnia. This could be explained because patients were not in advanced-stage disease and most patients were classified as RA-PD. According to several authors [28–31], EDS is associated with advanced-stage parkinsonism and longer disease durations, suggesting that severe and widespread brain damage predicts higher hypersomnia.

To our knowledge, this is the first study that analyzes individual symptoms of nocturnal sleep problems and hypersomnia over time and evaluates how clinical aspects of PD predict these disturbances. Specifically, time had a slightly positive effect on some symptoms, such as presenting less waking tremor over time, but this could be explained by the dopaminergic treatment. Although multiple cross-sectional studies assess nocturnal sleep problems and EDS in PD [6, 14, 24], few have investigated longitudinal changes, and no prior studies report long-term changes of individual sleep symptoms.

Regarding influence of motor symptoms on nocturnal problems and EDS over time, we found that higher motor impairment and age predicted more distressing hallucination at night, more nocturnal problems related to urine, and more tiredness after waking [17, 32, 33]. According to Mondragon-Rezola et al. [33], sleep fragmentation is the most frequent sleep disorder in PD. The difficulty to start or maintaining sleep can be a primary disorder, which is associated with PD itself, and is a conciliation insomnia, but it could also be secondary to other causes such as motor symptoms. The characteristic motor symptoms, such as tremor, rigidity, or akinesia, although usually decrease during sleep, may not disappear completely, altering the quality of sleep. In fact, night-time motor symptoms generally appear due to wearing “off” effect of dopaminergic therapy, and therefore can be related to urine incontinence [17]. However, this can be improved with continuous dopaminergic stimulation throughout the night, by administering nightly doses or long-acting dopaminergic doses, such as transcutaneous rotigotine or delayed-release ropinirole [10, 33]. Also, stimulation of the subthalamic nucleus has been shown to reduce sleep fragmentation, motor symptoms improvement and reduction of the antiparkinsonian drugs dose [34]. On the other hand, according to Goetz et al. [32], hallucinations seem to be related to higher motor scores and are associated with severity of vivid dreams or nightmares. Hallucinations could be dream-like phenomena related to REM and non-REM disruptions, anatomically associated to sleep-related nuclei including the reticular activating system and the parapontine nucleus [32].

Concerning EDS, higher motor impairment predicted more hypersomnia while watching TV, sitting inactive or active. According to several authors [17, 31, 35], EDS is associated with most of the PD motor and non-motor symptoms, and sleepiness is more likely to affect patients with advanced PD, since these patients presented shorter mean sleep latency compared to early PD and controls. This sleep latency was related to disease duration and motor impairment, but some authors did not find any association between EDS and motor symptoms severity [17, 30, 36]. According to Höglund et al. [36] and Gjerstad et al. [18], EDS in patients with PD is a complex non motor symptom with multifactorial underlying pathophysiology and it could be also related to other disorders such as obstructive sleep apnea [37] or narcolepsy [3].

Dopaminergic agonist treatment in monotherapy was associated with less painful posturing of extremities at wake up, suggesting that dopaminergic agonist was related to less nocturnal sleep problems [18, 38]. We found the contrary effect of medication on daytimes sleepiness. Agonist treatment was associated with more hypersomnia while disease duration and L-dopa in monotherapy were associated with less daytimes sleepiness. Therefore, for long-term EDS outcomes, L-dopa in monotherapy affected positively and dopaminergic agonist treatment affected negatively [17, 18, 24, 33]. According to our results and others findings, the agonist treatments can have beneficial effects, such as motor symptoms during sleep, or, conversely, produce or aggravate sleep disorder, contributing to fragmented sleep and reduced REM sleep or worsened EDS [18, 33, 38]. Consequently, the PD treatment could be responsible for certain sleep disorders. The effects of medication on sleep are complex, depending on the doses and the dopaminergic receptors involved, by mechanisms that have not been completely unraveled.

Sex also influences sleep problems in PD [17, 31], being men more likely to be sleepy and presenting more subjective EDS than women, even after controlling for L-dopa equivalent daily dose [17, 39]. Males presented less nocturnal sleep problems than females over time, but more EDS symptoms [40].

In summary, sleep disruptions in PD are common due to multiple factors including the neurodegenerative process of the disease itself. Furthermore, as PD is marked by dopamine dysfunction and degeneration of the dopaminergic neurons, dopamine network is also implicated in several major sleep disorders [3]. Sleep disturbances are also associated with impairment of other non-motor aspects [36], such as depressive symptoms, lower quality of life, increased fatigue, and poorer cognition [3], and can even predict dementia [41].

One of the major limitations of this study is that data are derived from a registry of PD patients during the clinical routine, and the patients were RA-PD type. A second limitation is that sleep problems were assessed prospectively when patients came to the hospital, without a set time for a follow-up visit, although mixed models controlled this factor. A third and fourth limitation is the lack of a control group and not including data about neuropsychiatric symptoms as depression. Future studies should include a cohort of healthy controls to assess the evolution of sleep disturbances in healthy population, and to take into account the neuropsychiatric and neurophysiological data. Finally, we only analyzed the effect of dopaminergic therapy, but the effect of other drugs, such as hypnotics or anti-depressives, would be recommended in forthcoming studies.

## Conclusions

In conclusion, this clinical study presented how sleep disturbances, nocturnal problems and daytime sleepiness, changed over time and how age, disease duration, motor impairment, dopaminergic treatment and sex were associated with these changes. We found that motor symptoms, disease duration, age, dopaminergic treatment, and male sex influenced on sleep problems (nocturnal and EDS) along time. The results highlighted that agonist treatment are associated with worse EDS but with better nocturnal sleep problems outcomes over time, whereas L-dopa in monotherapy could be related to less daytime sleepiness, and therefore significantly affect the quality of life of patients with PD.

## Supporting information

S1 Dataset (XLSX)
